# Visual perception of repaired cleft lip scarring face associated with different malocclusions via eye-tracking

**DOI:** 10.34172/joddd.2022.008

**Published:** 2022-05-29

**Authors:** Lara Karolina Guimarães, Gil Guilherme Gasparello, Matheus Melo Pithon, Mohamad Jamal Bark, Sergio Luiz Mota Júnior, Orlando Motohiro Tanaka

**Affiliations:** ^1^Department of Orthodontics, Post-Graduation Program, School of Life Sciences, Pontifícia Universidade Católica do Paraná, Curitiba, Brazil; ^2^Department of Orthodontics, Dental School, Southwest Bahia State University, Jequié, Bahia, Brazil; ^3^Department of Orthodontics,Dental School,Juiz de Fora Federal University, Juiz de Fora, Minas Gerais, Brazil

**Keywords:** Cleft lip scarring, Eye-tracking, Cleft lip and palate, Esthetics, Perception, Malocclusion

## Abstract

**Background.** This study aimed to evaluate the visual facial perception in response to scars associated with repaired cleft lip (CL) on a male adolescent patient, as assessed via eye-tracking.

**Methods.** Index of orthodontic treatment need (IOTN) malocclusions, grades 1, 5, and 8 were added to the frontal view facial image of an adolescent male model showing asymmetries of the nose and upper lip after CL surgery using the software Photoshop CS5^®^ software. The eye movements of 91 laypeople observers were tracked by an Eye Tribe infrared sensor connected to OGAMA^©^ software. A Kruskal–Wallis test was used to identify differences in total fixation time and time until the first fixation for the areas of interest. A visual analog scale (VAS) of attractiveness was also used in the study. Statistical analysis was performed adopting a significance level of *P*<0.05.

**Results.** The area of interest (AOI) were found to be the mouth and teeth, which were more focused on gazed at than any other area, regardless of the grade of IOTN. For observers of different ages, there were significant differences in the time until the first fixation on the scar of the repaired CL region for IOTN grade 1 (*P*=0.007). Images showing IOTN grade 1 repaired CL regions received the highest VAS scores. The older the age, the greater the tendency to give a higher VAS score for the same malocclusion.

**Conclusion.** The presence of a CL scar on the upper lip did not attract the eye of laypeople observers of different ages, regardless of the degree of malocclusion in the non-smile image. The age of the observers did influence the perception of attractiveness, with older observers giving higher scores than younger ones. As the severity of the malocclusion increased, they were found to be less attractive.

## Introduction

 Cleft lip (CL), with or without cleft palate (CLP), is the most common congenital malformation of the head and the third most common congenital defect.^1^ CL is approximately twice more common in men than women.^2^ Clinical management of CL is an ongoing and unique challenge in maxillofacial plastic surgery, the goals of which are to repair and achieve normal facial appearance, nutrition, speech, and hearing without significantly affecting the child’s ultimate facial and psychosocial development.^1^

 The cleft deformity often affects the patient’s facial appearance with scar tissue formation,^3^ and can be seen as a facial asymmetry, mainly in the nasolabial area.^4^ This condition can induce a social stereotype in first impressions.^5^ Children with CLP are often rejected by their peers. Any reference to the cleft in casual social encounters can cause anxiety, anger, shame, and anguish.^6^ Elementary school-age children with a CL have lower self-esteem, perceive themselves as less accepted by their peers, and are sadder and angrier red to children without a cleft.^7^

 Professionals are more familiar with the esthetic outcomes and difficulties of treating patients. The disparity between what is achievable by surgery and what is expected by laypeople may be a source of their dissatisfaction with post-surgery facial appearance.^8^ Laypeople and professionals rate the facial appearance of individuals with repaired complete unilateral or bilateral cleft lip and palate (UCLP and BCLP, respectively) similarly when viewing full facial images; however, differences in perception exist between healthcare professionals and laypeople. The discrepancies between the professional groups could be attributed to different treatment modalities and protocols.^9^

 There is considerable evidence that individuals with cleft lip and palate suffer the psychosocial consequences of their facial appearance despite advanced cosmetic surgery,^10^ but data on how their faces are perceived by others (e.g., data on the eye movements of individuals of different age groups when viewing post-surgery facial images) are lacking. Visual perceptions of the faces of adolescents with a bilateral repaired CL are viewed differently depending on the gender and age of the observer, and such information is important for optimal multidisciplinary management. It is unknown whether laypeople are more or less critical than professionals when rating the facial appearance of patients with repaired CLP.

 Therefore, this study aimed to evaluate and analyze the visual perception and judgment of attractiveness of different categories of observers by determining, through eye-tracking and subjective testing, the impact of scars from CL repair on the facial esthetics of adolescents.

## Methods

###  Photographs

**Figure 1:**
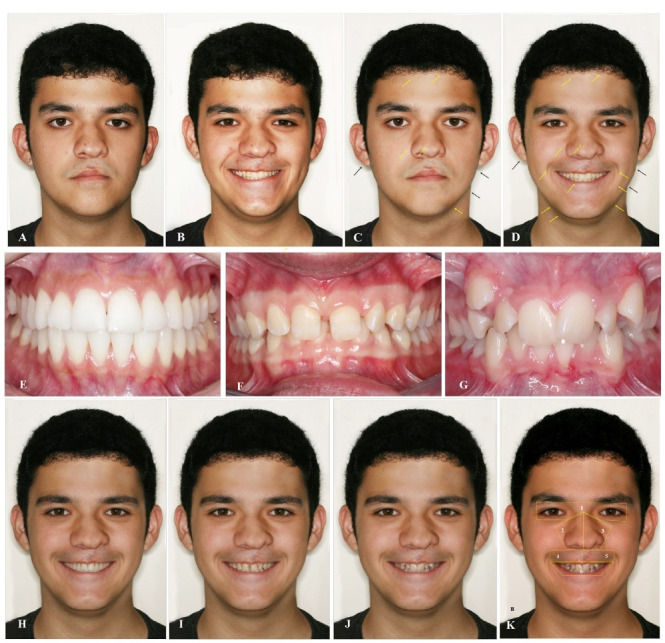
The photographs of the patient.

 Frontal-view facial images, one no-smiling and smiling, of an adolescent male patient showing asymmetries of the nose and upper lip after CL surgery, from a private office were used for this study. The photographs, shown in Figures 1A and 1B, respectively, were taken after rapid maxillary expansion and full brace orthodontic correction of a Class III malocclusion (Rebel XTI; Canon, Tokyo, Japan).

###  Data preparation

 Initially, the left hemi-face and hair (Figure 1A, B) were mirrored to generate a symmetrical face using Adobe Photoshop CS5^®^software (Adobe Systems Inc., San Jose, CA). The lower lip and facial right contour were mirrored with the left side Figures 1C, D arrows). The unilateral cutaneous scars on the upper lip and associated nasal asymmetry were maintained. Skin marks, pigmentations, facial hair, eyebrows, props, facial tattoo, extreme facial hair, or exotic hairstyle, and other features that could have interfered with the analysis of the images were removed.

 Images corresponding to index of orthodontic treatment need (IOTN) grades 1 (Figure 1E), 5 (Figure 1F), and 8 (Figure 1G) were edited and added to compose the smile images, forming Figures 1H–J.

###  Raters

 The sample size calculation was carried out following the heterogeneous population of Paraná state, Brazil adopting an infinite population, with a confidence level of 95% and a margin of error of 10%, and concluded that 91 people would be needed for the study.

 Participation was voluntary, recruited in 5 countryside cities in the state of Paraná, Brazil, and all observers gave written informed consent and affirmed they had good vision and were not taking any medication that might interfere with their cognitive or motor skills. They agreed to the exclusion criteria for the study: neurological alterations, recent use of drugs or alcohol, and taking medication that could interfere with cognitive abilities. Information including gender, age, and profession was provided by each participant because it was required by the software interrogatories, which needed to be answered completely to move to the next step in the eye-tracking process.

 The 91 laypeople observers approached for the study were separated into 3 age groups: A (15–44 years); B (45–59 years); and C (60 years or older). The sample consisted of 44 men and 47 women; 45 (50%) of the sample were graduates from higher education and 45 (45%) were not university educated.

 The observers were asked to evaluate images about which they had been given no additional information. They were informed only that the study concerned the perception of facial esthetics and that the images would be shown consecutively on the monitor. They were then seated comfortably in a quiet room at a distance of 75cm from a 17-inch high-resolution (768 × 1366 pixels) monitor (Dell P2317H; Dell Inc., Round Rock, TX) the images were projected vertically at true size, with an unobstructed view.

 TheEyeTribe© hardware (The Eye Tribe ApS, Copenhagen, Denmark) was used for eye tracking, in conjunction with OGAMA© 5.0 software (OpenGazeAndMouseAnalyzer), with the mouse image removed from the monitor so as not to interfere with data capture.

 For data collection with OGAMA© software, 4 areas of interest (AOIs) were delimited: (1) eyes; (2) right nose; (3) left nose; (4) upper lip, and (5) teeth with lower lip (Figure 1K). The heatmaps, fixation point maps, total fixation time, and direction transition values were all generated or measured automatically within these areas and the raters did not see the AOIs.

 The system was calibrated on a per-subject basis at the beginning of the experiment. The eye-tracking procedure started with a calibration sequence. To verify that the eye tracker was able to accurately capture each rater’s eye movements, the OGAMA^©^ software was calibrated for each participant, which involved following a ball with the eyes without moving the head to identify which raters were suitable and unsuitable for the study, with “excellent” and “good” constituting acceptable results and “poor” and “redo” serving as excluding factors.

###  Visual analog scale 

 After viewing all photographs for eye-tracking, the observers evaluated all images for a visual analog scale (VAS) of 0 to 100, with 0 denoting complete disagreement, 50 denoting neutrality, and 100 denoting complete agreement. This scale had a sliding bar, the position of which could be adjusted by the evaluator. The corresponding numerical value was saved in Microsoft PowerPoint 2010 (Microsoft, Redmond, WA) for the tabulation of the data.

###  Statistical analysis

 The results obtained from the eye-tracking and VAS were tabulated using Microsoft Excel 2019 Version 16 software (Microsoft Inc., Redmond, WA) and analyzed using SPSS Version 25 (Statistical Package for the Social Sciences software; SPSS Inc., Chicago, IL).

 The group of images with different IOTNs, and the group of observers, divided by age, were defined as independent variables for the study, while the areas of interest and VAS scores were defined as the dependent variables.

 The Kruskal–Wallis test was applied to evaluate the images about the VAS. Levene’s homogeneity test was applied to identify homogeneous or heterogeneous populations. As for the first fixation time and complete fixation time, the Kruskal–Wallis test for independent samples was applied to identify differences. The significance level adopted for this study was *P* = 0.05.

## Results

**Figure 2:**
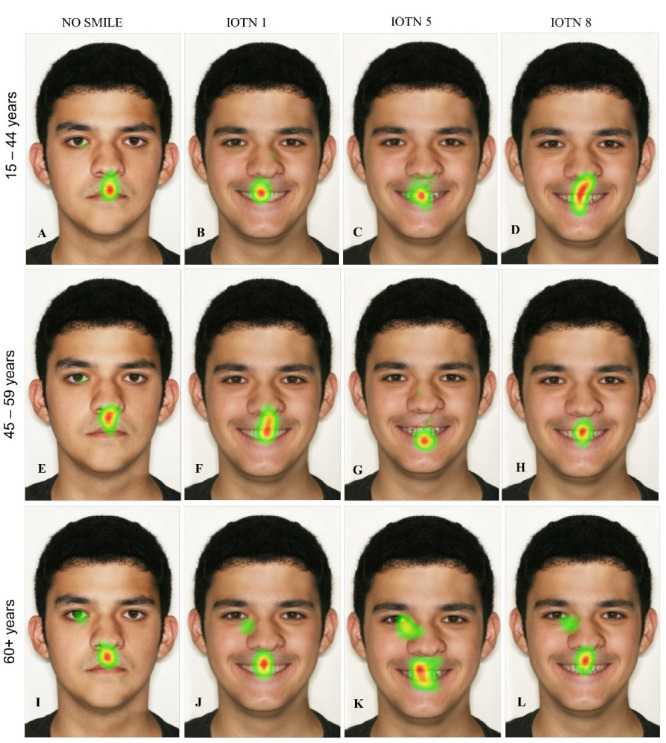
The heat map of the eye tracking is superimpoed on the photographs.

 Eye-tracking generated heatmaps for the images combining No Smiling and IOTN Grades 1, 5, and 8, which were divided into groups based on the raters’ ages (Figure 2). The pattern of visualization for the No Smiling image indicated that the gaze point centered mainly on the scarring. Regardless of the IOTN grade, the focus of the observers were not on the scarring area, but the mouth, and teeth. In the Elders group, there was also a focus on the region between the right eye and nose, for all images.

 As shown in Figure 2, age groups 15–44 and 45–59 show a predominance of fixations in the upper third of the face for the No Smiling and IOTN grade 1 images. For the IOTN grade 5 and 8 images, however, there are more fixations in the lower third of the face. For the 60+ age group, fixations are proportionally distributed in the upper and lower thirds of the face, regardless of the image presented.

 Regarding the eye eye-tracking different age groups, as shown in Table 1, there was no statistical difference in the AOIs contrasting with IOTNs for complete fixation time (*P* > 0.05), showing the repaired cleft lip scarring did not attract the eye, regardless of the malocclusion. Time until the first fixation also showed no statistical difference regardless of IOTN grade (*P* > 0.05).

**Table 1:** Mean of complete fixation time at eye, left nose, right nose, upper right lip, upper left lip and mouth, and respective time until first fixation, mean of VAS, and p-value (SIG) for Kruskal–Wallis test regarding different age groups and IOTN 1, IOTN 5 and IOTN 8. and IOTN 1, IOTN 5 and IOTN 8

**Age-IOTN**	**Complete fixation time at eye**	**Complete fixation time at left nose**	**Complete fixation time at right nose**	**Complete fixation time upon right lip**	**Complete fixation time upon left lip**	**Complete fixation time at mouth**	**Time until 1st fixation in eye**	**Time until 1st fixation in left nose**	**Time until 1st fixation in right nose**	**Time until 1st fixation in upon right lip**	**Time until 1st fixation in upon left lip**	**Time until 1st fixation in mouth**	**VAS**
	Mean (SD)	Mean (SD)	Mean (SD)	Mean (SD)	Mean (SD)	Mean (SD)	Mean (SD)	Mean (SD)	Mean (SD)	Mean (SD)	Mean (SD)	Mean (SD)	Mean (SD)
Serious young adults	786.00 (287.95)	1291.75 (384.06)	399.50 (601.48)	1024.50 (1024.50)	633.12 (264.44)	1119.80 (442.31)	1619.00 (1501.20)	1789.75 (1670.90)	1632.00(2308.00)	999.50(1762.23)	1456.75(1507.08)	1279.00(1075.74)	52.03 (3.94)
Serious middle age adults	471.43 (243.36)	832.25 (384.06)	417.00 (601.48)	599.50 (599.50)	799.80 (334.49)	625.00 (571.02)	2393.14 (1811.02)	1990.25 (1506.38)	950.00 (70.71)	33.25 (66.50)	1225.60 (861.48)	1661.67 (1709.17)	54.93 (4.42)
No Smile eldfers	1385.67 (371.74)	1187.33 (443.47)	1146.00 (380.41)	838.60 (838.60)	1065.67 (305.35)	465.00 (989.03)	1321.67 (2202.30)	1954.33 (1651.90)	619.60 (1118.11)	753.20 (709.66)	738.67 (1186.75)	367.00 (.)	58.70 (4.79)
Significance	0.289	0.788	0.935	0.875	0.419	0.867	0.476	0.974	0.621	0.188	0.551	0.495	0.450
IOTN 1 young adults	649.17 (262.86)	2148.00 (543.14)	2181.00 (601.48)	474.00 (474.00)	350.00 (528.88)	1470.83 (403.77)	1843.50 (1494.58)	400.00 (565.68)	.00 (.)	1848.50 (2023.93)	4531.00 (377.59)	1049.50 (1248.08)	62.18 (3.94)
IOTN 1 middle age adults	387.25 (227.64)	389.33 (443.47)	966.00 (601.48)	225.25 (225.25)	634.71 (282.70)	973.78 (329.68)	2560.50 (1436.77)	977.00 (717.64)	2615.00 (212.13)	1032.50 (1595.73)	1779.57 (713.02 )	1106.67 (1087.22)	63.00 (4.42)
IOTN 1 elders	522.00 (287.95)	532.50 (543.14)	299.60 (380.41)	1315.75 (1315.75)	349.75 (373.97)	1131.17 (403.77)	1298.40 (1443.54)	3197.00 (1600.89)	1985.20 (1544.89)	407.75 (370.70)	633.00 (151.78)	1266.17 (1452.57)	66.00 (4.79)
Significance	0.535	0.107	0.108	0.118	0.247	0. 976	0.294	0.107	0.158	0.629	0.007*	0.937	0.587
IOTN 5 young adults	233.00 (321.93)	522.33 (443.47)	441.50 (425.31)	711.33 (711.33)	1565.75 (373.97)	632.00 (373.82)	2365.00 (1553.43)	1665.33 (1240.85)	2955.50 (1827.76)	221.67 (194.87)	1182.25 (1141.13)	600.29 (769.93)	40.64 (4.00)
IOTN 5 middle age adults	150.00 (455.28)	942.67 (443.47)	888.00 (491.11)	409.00 (429.39)	1038.60 (334.49)	1314.73 (298.20)	3730.50 (1272.08)	1920.00 (1321.23)	1666.00 (1515.88)	474.75 (743.69)	1525.80 (776.31)	999.36 (1228.21)	43.33 (4.42)
IOTN 5 elders	810.67 (262.86)	545.00 (443.47)	640.75 (425.31)	773.75 (429.39)	1144.17 (305.35)	786.80 (442.31)	1632.17 (1688.10)	1498.33 (1321.55)	225.00 (159.81)	474.50 (691.66)	1659.33 (1794.36)	406.00 (452.56)	44.33 (4.69)
Significance	0.052	0.561	0.431	0.561	0.778	0.498	0.362	0.957	0.087	0.971	0.879	0.810	0.629
IOTN 8 young adults	1299.00 (455.28)	211.67 (443.47)	1000.00 (850.62)	1482.50 (607.25)	350.00 (528.88)	1398.50 (403.77)	.00 (.000)	155.33 (269.04)	733.00 (.)	1000.00 (377.59)	2765.50 (566.39)	1182.50 (1430.92)	32.97 (3.94)
IOTN 8 middle age adults	133.00 (643.87)	167.00 (543.14)	391.25 (425.31)	415.50 (607.25)	216.50 (373.97)	906.78 (329.68)	.00 (.)	2514.50 (211.42)	1374.25 (1622.71)	4431.00 (374.77)	1523.50 (1782.25)	921.11 (967.09)	37.07 (4.42)
IOTN 8 elders	1299.50 (455.28)	310.67 (443.47)	1454.33 (491.11)	1450.00 (429.39)	499.67 (431.83)	1094.17 (403.77)	1416.00 (2002.53)	2020.67 (1849.95)	644.00 (558.70)	1848.00 (1958.39)	2753.67 (1585.47)	1576.67 (1444.48)	44.17 (4.79)
Significance	0.368	0.228	0.068	0.264	0.604	0.643	0.472	0.233	0.950	0.135	0.760	0.821	0.130
Significance for all groups	0.108	0.113	0.323	0.491	0.102	0.993	0.108	0.113	0.323	0.491	0.102	0.993	0.000*

Notes: Means are noted in miliseconds (ms) The line «significance» correspond for each upper collum, separeted by Serious/IOTN grade. The line «significance for all groups» correspond for all raters, without Serious/IOTN grade division. (*) P < 0.05

**Figure 3:**
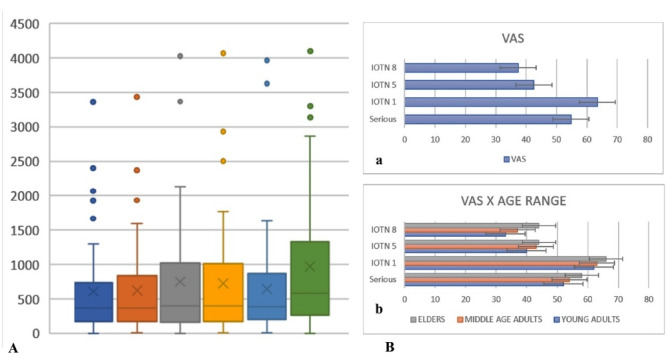
Visual analoge scale results.

Figure 3A presents a box plot showing the quartiles distribution, median, and outlier values of AOI fixation time.

 Concerning attractiveness, IOTN grade 1 images received high VAS scores (Figure 3B). Table 1 shows there was a statistically significant difference in VAS values for age groups and IOTN grade (*P* < 0.001). All age groups showed the same order for levels of VAS, suggesting that the older the age of the rater, the greater was their tendency to give higher VAS scores for the same malocclusion or No Smiling image (Figure 3B).

## Discussion

 The present study used eye-tracking technology to evaluate how laypeople of different ages rated a male patient with a scar from cleft lip treatment. Heatmaps and dot maps were used to visualize the raters’ viewing patterns when looking at images of smiling and non-smiling faces with cleft lip repair scars of three different IOTN grades.

 Subjective evaluation of cleft lip scarring can be affected by methodological approaches, professional experience, and stimulus.^3^ Eye-tracking methodology, which offers objective results, was the method chosen for this study, and as the CLP is more common in males,^2^ a male model was used to compose the present study.

 Facial symmetry is a fundamental goal of plastic surgery and is commonly regarded as a key component of human attractiveness.^11,12^ However, artificially generated, perfectly symmetric faces appear unnatural. Thus, some degree of facial asymmetry is attractive and is inherent in any face but, in excess, it is unnatural and unattractive and correlates with a decline in well-being.^13,14^

 The heatmap visualizations showed that raters focused mainly on the CL area of the No Smiling images and, when a smiling image was shown, they tended to look at the teeth and mouth area, regardless of the malocclusion. Heatmaps showing the patterns of raters from the 15–44 and 45–59 age groups indicated more fixations in the upper third of the face in No Smiling and IOTN grade 1 images. As the IOTN grade increased, fixations tended to predominate in the lower third of the face.

 The 60+ age group did not show a common pattern on heatmaps. Perhaps people in this age group place less importance on esthetic characteristics than younger people. Karp et al^15^ found a difference in the way older children directed their gaze to secondary cleft lip scarring, especially noting an increased gaze time on the CL than was measured for younger children.

 The presence of unilateral CL was not a reason for laypeople aged from 25 to 35 years to have a first fixation or highest fixation time in the cleft region in an individual with the cleft healed in rest and smiling photographs.^16^ In the present study, young adults focused the eye to scar region for non-smiling photographs and to teeth when the patient was shown smiling.

 There was no significant difference in complete fixation time, regardless of the age group of the raters, but when time until the first fixation at AOIs was evaluated, there was a difference for IOTN grade 1 for left lip, which suggests that the rater’s age affected the time until the first fixation in the scar region for a CL treated individual with a good occlusion.

 Laypeople consider that people who have had treatment of a CLP are more affected in their professional life than in social interactions.^9^ In this study, laypeople evaluated images of a male model with a scar from CL treatment and graded attractiveness by use of VAS. There was shown to be a statistically significant difference (*P* < 0.001) in VAS values given for No Smiling or malocclusion images by raters in different age groups. IOTN grade 1 images were considered the most attractive, followed by the No Smiling image, IOTN grade 5, and IOTN grade 8. These findings suggest that light malocclusion makes people’s CL scars more attractive when smiling than when not smiling. As the severity of their malocclusion increases (e.g., IOTN grades 5 and 8), such people are perceived to be less attractive. These findings should encourage clinicians and cleft lip patients to seek orthodontic treatment as soon as indicated, because of the importance of esthetics and attractiveness for a patient’s self-image in a social environment.

 The photographs of the patient evaluated in the present study, who was well-treated for CL, received median fixation time for scar region (left lip and left nose) with minor time than another side without scars, with a major number of raters for fewer times. These findings suggest that CLP fissures must receive attention from orthodontists and families for treatment to allow the patient to live with high quality of life.^5-7^

 Esthetics, functional improvement, and social external influences are motivating factors and common reasons for pursuing orthodontic care. Studies have shown that only 34% of patients are satisfied after orthodontic treatment,^17^ and patients with unrealistic expectations are prone to dissatisfaction with treatment.^18^ For instance, Michelogiannakis et al^19^ found that patients expected fewer checkups and diagnoses, less discussion about treatment at the initial visit, more dietary restrictions, and less improvement in smile esthetics and social confidence with orthodontic treatment than parents. Therefore, before beginning treatment, clinicians must talk with patients and parents about all diagnostics, treatment planning, phases, objectives, possibilities, and realistic achievable goals regarding the orthodontic treatment and retention period, and such discussions should be recorded.

 Even when viewing facial images showing CL scars, laypeople raters showed lower average times for the first fixation in the mouth area than in the scar region (except for elders for IOTN grade 1). Laypeople and professionals alike rate the facial appearance of patients with CLP consistently lower than for noncleft individuals.^20^ However, when assessing the facial appearance of individuals with clefts, there are conflicting opinions between laypeople and professionals. Some studies have reported professionals as being more critical,^21^ while others reported that laypeople are more critical.^8,20^

 Evaluations of postsurgical facial appearance in patients with CLP by laypeople and professionals have focused on cropped photographs of the isolated nasolabial region,^22,23^ while others have evaluated the full frontal facial image of patients with cleft lip and palate.^24,25^ The use of cropped photographs may not be appropriate, as they do not indicate total facial harmony and may therefore be misleading. The esthetic outcome of the repaired CL should not be viewed in isolation but should be based on overall facial appearance as it is in orthognathic patients.^26^

 We have used the full-face photo and disagree with Valverde-Montalva et al.^27^ We decided to use full-face photographs (mainly unchanged, but with specific alterations) over perioral frontal photographs because the full-face view has fewer facial features that may generate distractions.

 In the assessment of “atypical” nasal and lip appearance outcomes compared to “typical” appearance outcomes after UCLP repair, when judged by professionals, patients with repaired UCLP, and laypeople, noses with a smaller nostril and lips containing a whistling deformity were perceived as poorer outcomes compared to the “typical” results. Professionals, patients, and laypeople agree when assessing these outcomes.^28^

 The clinical impact of a rating difference between laypeople and professionals could have an influence on decisions regarding secondary surgical procedures for patients with CLP. One possibility is that laypeople and professionals assess facial appearance similarly, and both agree further surgical intervention would be beneficial. The other possibility is that laypeople and professionals rate facial appearance differently. If laypeople are more critical, then the surgical team may need to manage expectations or discuss the possibility of further surgery. If, however, professionals are more critical, then the facial appearance outcome is likely to be accepted even though the professional may feel the result is suboptimal and could be improved.^26^

 This study emphasizes the importance for professionals to strive for a symmetrical nasolabial appearance outcome during primary lip closure and primary nasal correction, avoiding a smaller nostril, and especially a whistling deformity.^28^

 A possible limitation of this study is that this was a cross-sectional observational study and, thus, the results were from a specific point in time. However, we believe this to be an alternative to obtaining this kind of data. These findings should encourage clinicians and CL patients to seek orthodontic treatment as soon as indicated, because of the importance of esthetics and attractiveness for a patient’s self-image in a social environment. A better understanding of peer perception has the potential to guide future interventions concerning secondary CL scarring and other facial deformities in pediatric patients.

## Conclusion

 The presence of a CL scar on the upper lip did not attract the eye of laypeople observers of different ages, regardless of the degree of malocclusion in the non-smile image. The age of the observers did influence the perception of attractiveness, with older observers giving higher scores than youngers. As the severity of the malocclusion increased, they were found to be less attractive.

## Authors’ Contributions

 LKG, GGG and MMP:Conception and design of the work, Acquisition, analysis, or interpretation, Drafting the work. MJB, SLMJ and OMT: Writing the article, Critical revision of the article, Statistical analysis.

## Funding

 None.

## Ethical Approval

 Ethical approval for this study was obtained from the ethics committee of the Pontifícia Universidade Católica do Paraná. Reference number (3,729,413). Additionally, written informed consent was obtained from the patient for the clinical details and any identifying images.

## Competing interests

 The authors declare that they have no competing interests.
